# Genomic rearrangements and signatures of breeding in the allo-octoploid strawberry as revealed through an allele dose based SSR linkage map

**DOI:** 10.1186/1471-2229-14-55

**Published:** 2014-03-01

**Authors:** Thijs van Dijk, Giulia Pagliarani, Anna Pikunova, Yolanda Noordijk, Hulya Yilmaz-Temel, Bert Meulenbroek, Richard GF Visser, Eric van de Weg

**Affiliations:** 1 Wageningen-UR Plant Breeding, Wageningen University and Research Centre, P.O. Box 16, 6700 AA Wageningen, The Netherlands; 2 Graduate School Experimental Plant Sciences, Wageningen University, Wageningen, The Netherlands; 3 Department of Agricultural Science, University of Bologna, Viale Fanin 46, 40127 Bologna, Italy; 4 The All-Russian Research Institute of Horticultural Breeding (VNIISPK), p/o Zhilina, Orel, Russia; 5 Department of Bioengineering, Ege University, 35100 Izmir, Bornova, Turkey; 6 Fresh Forward Breeding B.V, Wielseweg 38a, Eck en Wiel, The Netherlands

**Keywords:** Genomic rearrangement, Inversion, *Fragaria*, Polyploid, Haplotype, Homozygosity, MAS, Selection, Breeding signature

## Abstract

**Background:**

Breeders in the allo-octoploid strawberry currently make little use of molecular marker tools. As a first step of a QTL discovery project on fruit quality traits and resistance to soil-borne pathogens such as *Phytophthora cactorum* and *Verticillium* we built a genome-wide SSR linkage map for the cross Holiday x Korona. We used the previously published MADCE method to obtain full haplotype information for both of the parental cultivars, facilitating in-depth studies on their genomic organisation.

**Results:**

The linkage map incorporates 508 segregating loci and represents each of the 28 chromosome pairs of octoploid strawberry, spanning an estimated length of 2050 cM. The sub-genomes are denoted according to their sequence divergence from *F. vesca* as revealed by marker performance. The map revealed high overall synteny between the sub-genomes, but also revealed two large inversions on LG2C and LG2D, of which the latter was confirmed using a separate mapping population. We discovered interesting breeding features within the parental cultivars by in-depth analysis of our haplotype data. The linkage map-derived homozygosity level of Holiday was similar to the pedigree-derived inbreeding level (33% and 29%, respectively). For Korona we found that the observed homozygosity level was over three times higher than expected from the pedigree (13% versus 3.6%). This could indicate selection pressure on genes that have favourable effects in homozygous states. The level of kinship between Holiday and Korona derived from our linkage map was 2.5 times higher than the pedigree-derived value. This large difference could be evidence of selection pressure enacted by strawberry breeders towards specific haplotypes.

**Conclusion:**

The obtained SSR linkage map provides a good base for QTL discovery. It also provides the first biologically relevant basis for the discernment and notation of sub-genomes. For the first time, we revealed genomic rearrangements that were verified in a separate mapping population. We believe that haplotype information will become increasingly important in identifying marker-trait relationships and regions that are under selection pressure within breeding material. Our attempt at providing a biological basis for the discernment of sub-genomes warrants follow-up studies to streamline the naming of the sub-genomes among different octoploid strawberry maps.

## Background

Cultivated strawberry (*Fragaria x ananassa*) is an important soft fruit species that is grown worldwide. Strawberry is a vegetatively propagated outbred species derived from the hybridisation of two new world species (*Fragaria chiloensis* and *Fragaria virginiana*) in the 18th century [1]. As a member of the Rosacaea family, it shares ancestry with a variety of important food and ornamental crops such as apple, pear, peach and rose. Despite its economic importance and membership in a well-studied family, strawberry breeding to date rarely incorporates the use of molecular marker resources due to its complex, allo-octoploid genetic composition [2]. Because of this complexity, there are only a limited number of studies where clear marker-trait relationships for major genes/QTLs were identified ([3-8]).

The first comprehensive molecular genetic maps in strawberry were developed for the diploid wild species *Fragaria vesca*[9-12]. This effort culminated in the completion of the draft sequence of the diploid *Fragaria vesca* clone ‘Hawai 4’ in late 2010 [13], which provided the rosaceous community a highly valuable tool for further genomic research.

Soon after the first genetic map of diploid strawberry was published, similar studies were initiated for the octoploid strawberry, resulting in the completion of several (partial) genetic maps [6,14-22]. These mapping studies also conclusively revealed that the octoploid strawberry showed genome-wide disomic inheritance [1,2] and could therefore be classified as a full allopolyploid.

The origins of the homoeologues (or sub-genomes) in allo-octoploid strawberry have not been studied as extensively as those of other allopolyploid crops such as bread wheat and Cotton [23,24]. Molecular genetic studies revealed that the chloroplast DNA of octoploid strawberry is most closely related to that of the diploid species *Fragaria vesca* (subsp. bracteata) [25,26]. In another study on nuclear genes, it was confirmed that part of the genome was clearly related to *Fragaria vesca*, and another part was related to the wild diploid *Fragaria innumae*, leading to the hypothesis that the octoploid genome originated from the fusing of unreduced gametes of two tetraploid species from quite distinct genetic backgrounds. To date, no convention exists for naming homoeologues, and none of the octoploid mapping studies have incorporated information on the origins of the different homoelogues in the naming of the linkage groups. For this reason, the assignment of homoelogue letters is not consistent between the different octoploid maps.

The need to obtain complete haplotype information from microsatellites utilised in polyploids motivated van Dijk et al. [27] to develop the MADCE (Microsatellite Allele Dose Configuration & Establishment) methodology for determining the allelic configuration of allopolyploid plant species [27]. This method essentially converts any allopolyploid genome into a diploid genome regarding the software and methodologies that can be employed for genetic analysis.

In this study, we created a highly comprehensive genetic SSR linkage map of the octoploid strawberry using MADCE. We used this map to differentiate homoeologues based on their efficiency in amplifying *F. vesca*-derived markers and to discover genomic rearrangements among the diploid sub-genomes (homoeologues). This map provides the genetic makeup of the two parental varieties and their levels of homozygosity and haplotype sharing. Finally, we made comparisons of the cultivated strawberry genetic map to the physical reference map of the wild diploid *F. vesca*.

## Methods

### Plant materials

**Figure 1:**
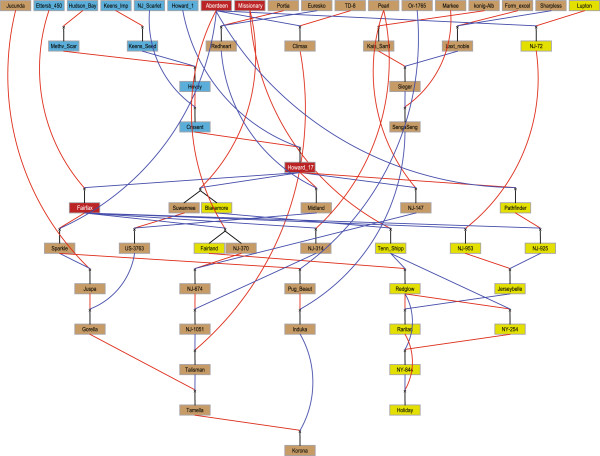
**Pedigree of mapping parents Holiday and Korona.** Red lines indicate maternal parents, and blue lines paternal parents. Yellow-green coloured parents are unique to Holiday, brown coloured parents are unique to Korona, and the red colour indicates the closest common ancestors for Holiday and Korona. Blue coloured individuals are ancestors or parents of the closest common ancestors of Holiday and Korona. This figure was drawn using Pedimap [28].

For the construction of a molecular marker linkage map, a subset of 92 seedlings from a cross between the strawberry cultivars ‘Holiday’ and ‘Korona’ was used. DNA admixture and possible outcrossings resulted in the removal of ten individuals, leaving a total of 82. The pedigree of Holiday and Korona is presented in Figure 1. Another F1 population of 133 individuals derived from a cross between ‘Elsanta’ and selection E1998-142 was used to confirm an inversion observed in Holiday x Korona. The mapping populations were created at and are maintained by the private breeding company Fresh Forward Breeding BV.

### DNA isolation

Genomic DNA was extracted according to a modified version of the Fulton et al. [29] mini-prep protocol. Briefly, 1 g of young, folded leaves were harvested. The leaves were freeze-dried and ground to powder in a 2 ml tube. To this tube, 700 μL of warm (65°C) containing 2% CTAB buffer was added, and the contents were mixed by vortexing and incubated for 10 min. Next, 700 μL of chloroform:isoamyl alcohol (24:1) was added. The mixture was centrifuged at room temperature at 10,000 g for 2 min. Next, 600 μL of the top phase was transferred to a fresh tube. Isopropanol (480 μL) was added, and the sample was mixed and then centrifuged at 10,000 g for 2 min at room temperature. The supernatant was discarded, and the pellet was washed with 500 μL of 70% ethanol, left for 2 min and then centrifuged at 10,000 g for 2 min. The supernatant was discarded by pipetting, and the pellet was resuspended in 400 μL of Tris EDTA. LiCl (135 μL, 8 M) was added to remove RNA and polysaccharides, and the mixture was incubated for 30 min at −20°C. After incubation, the mixture was centrifuged at room temperature at 10,000 g for 2 min, and the supernatant was transferred to a fresh tube. Isopropanol (320 μL) was added, and the mixture was incubated at −20°C for 30 min. The mixture was then centrifuged at 10,000 g for 5 min, and the supernatant was discarded. The pellet was then washed and centrifuged twice with 500 μL of ethanol (70%), and then the dried pellet was dissolved in 50 μL of TE.

### SSR markers

#### Origin

A total of 186 primer combinations from a variety of sources [6,10-12,15,18,22,30-43] were used for the construction of the linkage map. These primers were selected to obtain genome-wide coverage of 10-20 cM intervals with the least possible number of markers. The parameters considered were the length of the SSR repeat, the polymorphism level between our mapping parents and, when available, mapping information from previous publications. Complete information on these primer combinations can be found in Additional file 1: Table S1.

#### PCR

The first approximately 50 primer combinations used in this study were directly labelled with fluorescent dyes (6-FAM, NED or HEX). Subsequent PCR reactions were performed with indirect fluorescent labelling [44] using a universal 17 bp 5’ end tail sequence (AACAGGTATGACCATGA) on the forward primer, which matched a universal fluorescently labelled primer (6-FAM, HEX or ROX) [44]. All reverse primers had a GTTT tail [45] on the 5′ end to minimise stutter formation. The PCR mixture was composed of 1 X Goldstar PCR buffer, 0.05 μM unlabelled forward primer with a tail, 0.2 μM unlabelled reverse primer, 0.2 μM labelled universal primer, 0.3 U of Goldstar Taq polymerase (Eurogentec Nederland B.V., Maastricht, The Netherlands) and 10 ng of DNA in a total reaction volume of 20 μL. In the case of directly labelled primers, the same mixture was used except that the forward (directly) labelled primer and reverse primer were both present at 0.2 μM concentrations. The PCR conditions were one cycle at 94°C for 3 min followed by 35 cycles at 94°C for 30 s, 50°C for 30 s and 72°C for 2 min and a final extension cycle at 72°C for 10 min for both labelling methods.

#### Marker analysis

Fluorescently labelled amplicons were separated and detected using an ABI capillary automated sequencing platform (Initially ABI 3700 and later ABI 3730, Perkin Elmer Biosystems, Foster City, Calif.). The output from the ABI platform was analysed with either Genotyper 3.6 (ABI3700) or Genemapper 4.0 (ABI3730) software. Peaks corresponding to alleles were identified, and their bin ranges were defined. Next, for each sample, the software automatically identified the presence of alleles (peaks) and the area under the peak. Allele detection was checked manually and adjusted where necessary. The allelic data (size and area) for each individual (parents and progeny) were transferred to an Excel spread sheet. The analysis of the data followed the MADCE procedure for establishing allelic configurations in allopolyploid populations [27], which allowed us to estimate allele dose and to identify pairs of homologous alleles for each of the sub-genomes.

### Construction of linkage maps

The construction of linkage maps followed the same procedure as described by van Dijk et al. [27]. Briefly, during data analysis, the alleles were first assigned to homologous groups on the assumption that alleles shared between parents are most likely to originate from the same sub-genome, unless the data indicated otherwise. This approach allowed the definition of so-called bridge markers that link the two pairs of parental homologs. These markers are of type <hkxhk> and <efxeg> (Annotation of JoinMap® 3.0 and following versions for Cross Pollinating systems). Early data consistency checks were performed using allelic pairs as described previously by Sargent et al. [17]. Next, linkage maps were created for each parent separately using JoinMap® 4.0 (Kyazma B.V.) [46] applying the regression approach and Kosambi mapping function. These separate parental maps were compared to each other to match the parental maps belonging to the same homoeologue based on the already-identified <hkxhk>, <efxeg> markers similar to the method of Barrett et al. [47]. This information was used for increasing the number of integrated loci by converting <lmxll> and <nnxnp> markers from the same primer pair into <abxcd> markers, as well as for the validation of the previously identified <hkxhk> and <efxeg> loci. After this data check, integrated maps were created when possible. JoinMap® output was imported into Excel to check for possible genotyping errors (double recombinants) through a graphical genotyping approach [48]. Putative double recombination events were checked up to the level of the original ABI output. The map was regarded as final when the latest corrections did not result in new putatively erroneous double recombination events, which typically required one or two rounds of corrections. The map positions of loci where both parents were homozygous were added later by imputing them from the relative positions of their homoeologous loci. Primer pairs that amplified heterologous chromosomes were never imputed and were only shown on Linkage Groups (LGs) to which their amplicons mapped. The phase information generated by JoinMap® was used to establish the parental haplotypes. Drawings of the linkage maps were first created with the software packages MapChart [49] and later finalised in Adobe Illustrator CS5 (Adobe Systems, San Jose, CA).

### Denotation of sub-genomes

The assignment of a homoeologue letter (A, B, C or D) to a linkage group was based on the amplification efficiency of the *F. vesca*-derived primer pairs. The efficiency was expressed as the proportion of amplified alleles observed for all *F. vesca* primer pairs on a linkage group over the total numbers of alleles that were possible (amplified and null alleles).

Loci for which it was uncertain whether null alleles occurred (e.g., due to homozygosity on multiple LGs) were not included in the calculation for these LGs. Loci that amplified from heterologous chromosomes were only used for the efficiency calculations for the LGs on which they mapped.

### Comparative mapping

Physical map locations of the microsatellites used in this study were obtained by blasting the SSR primer sequences to the *F. vesca* pseudo-chromosome assembly v 1.1 [13,50]. When no clear hits were found, we used full-length sequences of the marker, when available. In the visualisations, the physical positions of the microsatellites in mega-base pairs were multiplied by three to better fit the scale of the genetic maps. The octoploid genetic map was represented by the homoeologue that had a good density of segregating markers and showed few inconsistencies in marker order with the other homoeologues. The diploid genetic map of Sargent et al. [51] was chosen as it had most primer pairs in common with our map. The genetic positions of the CO–and CX-series of markers [18], were imputed using data from a recent diploid genetic map [50]. The maps were completed in Mapchart [49] and finalised in Adobe Illustrator CS5.

### Estimation of homozygosity levels and haplotype sharing between parents

Holiday and Korona both have ancestors that occur multiple times in the known parts of their pedigree (Figure 1), due to which, part of their genomes are likely to be homozygous by descent. In addition, Holiday and Korona are likely to have shared haplotypes, as they have some ancestors in common. The theoretically expected level of homozygosity was derived from a numeric relationship matrix obtained with FlexQTL [52]. This matrix consists of doubled kinship coefficients. The observed levels of homozygosity and haplotype-sharing were estimated using our linkage map. For this map, we identified genetic regions that had multiple (3 or more) adjacent loci where the alleles were identical, within parents (for homozygosity estimation) or between parents (for haplotype sharing/kinship estimates). Because multiple adjacent loci were used, these identical by state (IBS) regions were assumed to be identical by descent (IBD). The genetic length covered by such regions was assessed and totalled. To calculate the homozygosity levels, we divided the genetic size of the homozygous stretches by the genetic size of the genome. Briefly, the linkage map-derived kinship coefficients were calculated using Gillois identity states [53] for each genomic region (in cM) and their associated Jacquard condensed coefficients of identity [54]. On our linkage map, several identity states can be distinguished. First, there are areas where no haplotype is found in common; these areas have a kinship coefficient of 0 (Gillois identity state Δ9). Next, areas with one haplotype in common between the parents have a kinship coefficient of 0.25 (state Δ8). Areas with two different haplotypes in common between the parents have a kinship coefficient of 0.5 (state Δ7). Areas where a haplotype is homozygous in one parent and the same haplotype is heterozygous in the other parent have a kinship coefficient of 0.5 (state Δ3 and Δ5). Areas where a haplotype is homozygous in both parents have a kinship coefficient of 1 (state Δ1). As an example, when a chromosome of 60 cM has identity states Δ8, Δ3, Δ1 and Δ9 on areas of 15, 10, 5, and 30 cM, respectively, its total kinship coefficient amounts to 0.23 ((15 cM*0.25 + 10 cM*0.5 + 5 cM*1 + 30 cM*0)/60 cM).

### Duplicated microsatellite analysis

To investigate the underlying causes of multi-locus targeting of microsatellites we performed a BLAST search of these sequences against the *Fragaria vesca* reference genome (cutoff value 1*E^−10^). We checked whether the reference genome annotation showed a transposable element identified by LTRHarvest [55] overlapping the location to which the marker was BLASTed. We then used 4 kb of flanking sequence from the most significant hit and performed a BLAST search against the nucleotide collection from NCBI to establish whether the microsatellite was present within a gene.

## Results

### Global mapping results

**Figure 2:**
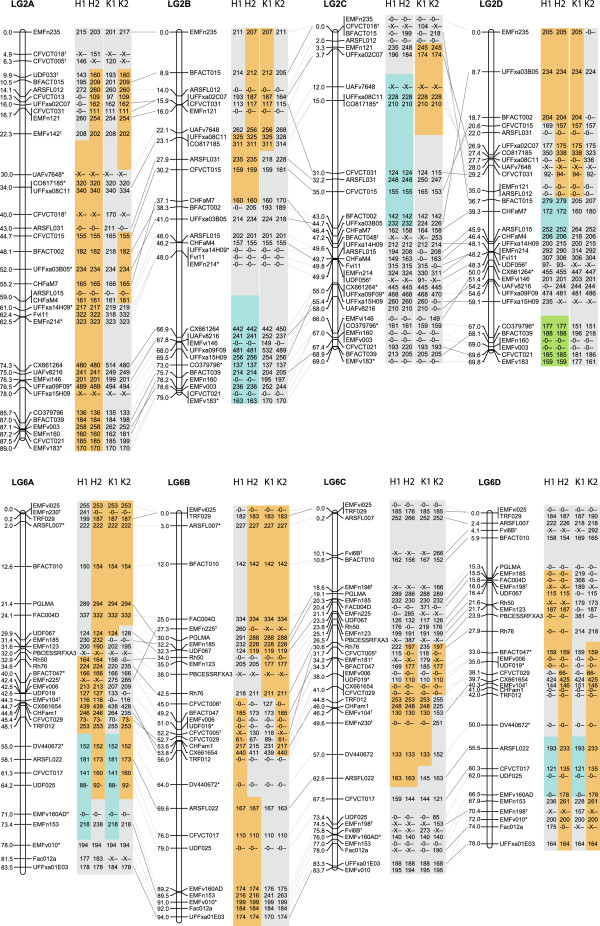
**Linkage maps for the 4 homoeologues of linkage groups 2 and 6 from the Holiday x Korona mapping population.** Allele sizes are given in the boxes next to the names of the SSR primer pairs. “X” signifies that no allele could be assigned, as some of the observed alleles could not be reliably scored. In the figure, “0” stands for a null allele. H1 indicates Holiday haplotype 1, K1 indicates Korona haplotype 1, etc. Regions highlighted in the same colour (within a homoeologue) indicate identical haplotypes. Dark grey lines connect homoeologous loci that segregated for both neighbouring homoeologues. For light grey lines, one or both of the homoeologous loci had its position imputed. An asterisk (*) indicates that the allelic composition can be switched between homoeologues due to multiple occurrences of homozygosity. A dagger (†) indicates a primer pair that amplifies on multiple heterologous chromosomes. The minimum resolution that still represents a single recombination event is 0.6 cM for regions in which both parents segregate and 1.2 cM where only one parent segregates. Any unit that is smaller occurred due to technical issues such as missing values, uninformative individuals and integration between parents. All LGs are available in Additional file 2: Figure S1.

A total of 186 SSR primer pairs were used to generate genetic linkage maps. They generated a total of 508 segregating loci, of which, 168 (35%) were bi-parental (<hkxhk>, <efxeg> and <abxcd> types). After splitting the bi-parental loci, the total number of loci segregating for Holiday amounted to 283 and for Korona to 393. The genetic map in its entirety is presented in Additional file 2: Figure S1. Linkage groups 2 and 6 are presented as an example in Figure 2. All 28 chromosome pairs of the strawberry genome were recovered. For just one pair (3C), the single parental maps could not be merged into an integrated map due to large differences in the recombination rates between the two parents for shared marker loci. Two additional, small linkage groups segregating only for Korona could not be unambiguously connected to the main body of their respective linkage groups (LG3A and LG3B). Apparently, their genetic distance was too large to connect these bottom groups with the nearest informative locus from the main body of the linkage group, at least with the given family size. The total length of the integrated maps sums up to 1846 cM, making the average genetic length of a linkage group 66 cM and the average marker density one in every 3.6 cM. This total length does not include the distance between the two bottom fragments of chromosome 3 and their respective top segments, and it also excludes the segments on the extremities of a linkage group where both parents were homozygous. Using the homoeologous positions of these homozygous marker loci, the estimated total genetic length of this map extends approximately 200 cM to a total of approximately 2050 cM.

### Denotation of sub-genomes

We denoted the four sub-genomes based on their level of sequence divergence from *F. vesca.* This divergence was determined by MADCE-derived genotype configurations using the proportion of amplified alleles over the total number of allowed alleles. The sub-genome with the highest efficiency (fewest null alleles) was assigned homoeologue letter A, and the sub-genome with the lowest efficiency (many null alleles) was assigned homoeologue letter D. The amplification efficiencies are shown in Table 1. LGs 1, 5 and 7 showed a stark contrast in *F. vesca* amplification efficiency between the first two homoeologues (A and B) versus the last two (C and D). In contrast, for LGs 3 and 6 and to a lesser extent LGs 2 and 4, the difference was mainly between homoeologue A and the other homoeologues. Another interesting phenomenon that was observed through the identification of null alleles was the presence of regions where several consecutive markers did not amplify any product. An example of this occurred in the centre of LG2B (Figure 2) where markers UFFxa14H09, Fvi11 and EMFn213, spanning more than one mega-base in physical distance, did not amplify any product. Other examples are observed on the distal parts of LG3B, −5B, −5C and 7C, as well as in the centre region of 6D (Additional file 2: Figure S1). These regions could constitute large deletions for specific homoeologues. Many other regions showed null alleles for one or two successive SSR loci. An increase in marker density for these regions may provide further evidence as to whether these results are indicative of true deletions or coincidental sequence divergences at the primer site(s).

**Table 1:** **Amplification efficiency of *Fragaria vesca*-derived SSR primer pairs**

***Linkage group***	***nr of vesca derived primer pairs***	***Amplified alleles/total alleles***	***Total vesca efficiency in %***
LG1A	13	48/52	92
LG1B	6	20/24	83
LG1C	5	10/20	50
LG1D	5	8/20	40
LG2A	11	42/44	95
LG2B	7	24/28	86
LG2C	8	27/32	84
LG2D	8	27/32	84
LG3A	13	46/52	88
LG3B	13	34/52	65
LG3C	12	27/48	56
LG3D	12	21/48	44
LG4A	6	23/24	96
LG4B	7	24/28	86
LG4C	4	13/16	81
LG4D	5	16/20	80
LG5A	7	28/28	100
LG5B	9	33/36	92
LG5C	8	22/32	69
LG5D	7	19/28	68
LG6A	10	38/40	95
LG6B	12	33/48	69
LG6C	11	30/44	68
LG6D	11	28/44	59
LG7A	6	19/24	79
LG7B	6	14/24	58
LG7C	4	5/16	31
LG7D	5	5/20	25

Differences in nr of primer pairs within a chromosome are caused by either heterologously amplifying primer pairs, or loci for which allele assignment was unclear (see footnote Figure 2).

### Genomic organisation of homoeologues: collinearity and re-arrangements

#### Overall collinearity

The overall collinearity between the homoeologues of a chromosome was very high. There were many small-scale divergences of only 1-2 cM (Additional file 2: Figure S1), but these divergences are likely due to mapping or scoring errors that were overlooked in the error checking, missing values or the presence of less informative <hkxhk> markers. In some cases, the differences in marker order were caused by the integration of the two parental maps. An example of this discrepancy can be observed in the order of markers EMFn185 and UDF067 (LG6, Figure 2). For LG6A, UDF067 occurred before EMFn185, whereas for the other homoeologues, it did not. The nearest locus for which both parents segregated was EMFn123. The distance from EMFn185 to EMFn123 was based solely on recombination events within Holiday, and the distance from UDF067 to EMFn123 was based solely on recombination events within Korona. A difference in the recombination frequency between the parents for that small region is the likely cause of the altered marker order.

#### Large rearrangement on chromosome 2

**Figure 3:**
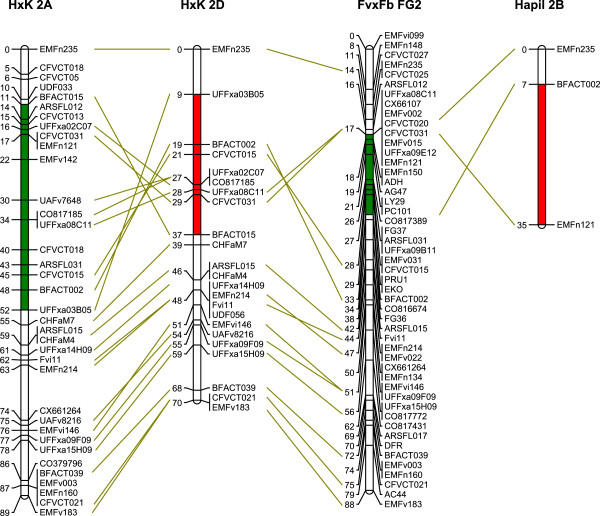
**Linkage maps demonstrating the inversion of LG2D.** On the left, LG2A of the octoploid Holiday x Korona is represented as a reference, and next to it is the LG2D of Holiday x Korona containing the putative inversion. To the right of LG2D is the diploid Fv x Fb map [51] On the far right, LG2B of the Hapil parent from Sargent et al. [17] is shown. The filled chromosome segments indicate the regions of interest. The segments with the same colour have the same orientation. The lines were drawn from locus name to the position instead of from position to position to facilitate the traceability of locus names.

We identified a major rearrangement in the marker order for LG2D (Figures 2 and 3), which an inversion that spans 28 cM (from marker UFFxa03B05 at 9 cM to BFACT015 at 37 cM) (Figure 3). Because both parents show the same inversion and because multiple segregating loci are located within this region, we believe this to be a genuine inversion.

A second putative rearrangement was found on LG2C and occurs within the homoeologous region of the former inversion (Figure 2). Here, a large gap appeared between marker loci UFFxa02C07 (at 4 cM) and CFVCT031 (at 31 cM), and close linkage was observed between CFVCT031 and ARSFL031, whereas for linkage groups 2A and 2B, these three markers showed the opposite pattern (Figure 2). Unfortunately, it was not possible to verify whether this result occurred due to an inversion, a translocation or simply a large difference in the recombination rate because the markers that are normally located between CFVCT031 and ARSFL031 were not informative, being either homozygous or impossible to discern for LG2C. In any case, the size of the rearrangement is smaller than that for LG2D, due to the difference in the position of the homoeologous loci for UFFxa03B05.

**Figure 4:**
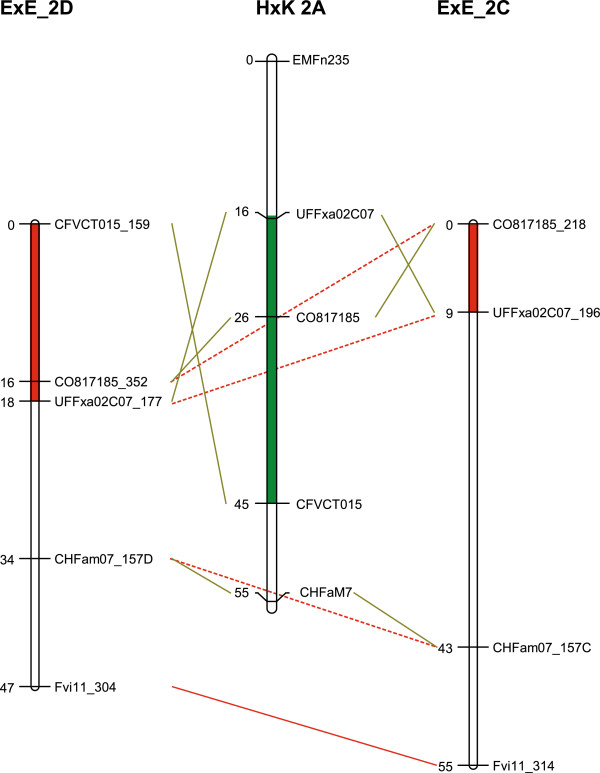
**Linkage maps supporting inversions on LG2 in different population.** Marker order of linkage groups LG2D and LG2C of mapping parent E1998-142 (from cross E1998-142 x Elsanta) and of the reference linkage group LG2A of Holiday x Korona.

The LG2 rearrangements were further examined in a second mapping population for which we had marker data available from a separate project, albeit at a lower marker density than that used for the Holiday × Korona map. The data confirmed the large inversion of LG2D in parent E1998-142 (Figure 4). Elsanta only had one marker segregating and could therefore not be used. For LG2C, we also found evidence for an inversion in E1998-142 (Figure 4). The evidence was not as strong as that for LG2D, however, because the two loci supporting the inversion were closely linked, and one of these loci (UFFxa02C07) was an <hkxhk> type marker, which are usually less accurately positioned due to hk progeny being uninformative for mapping. We re-examined previously published maps to support the existence of these rearrangements. The only indication for the occurrence of this inversion was in the octoploid map of Sargent et al. [16,17] for LG2B (which they later called LG2D) in cultivar ‘Hapil’ (Figure 3). It is likely that this linkage group matches our LG2D.

### Homozygosity & heterozygosity

**Figure 5:**
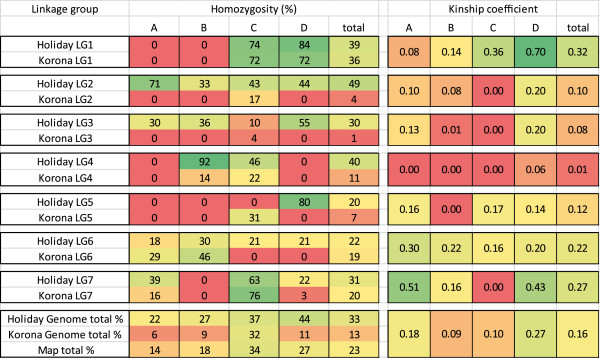
**Homozygosity and kinship coefficients per linkage group. A,B,C** and **D** stands for the different homoeologues of a chromosome.

The level of observed homozygosity in the mapping parents is shown in Figure 5. The genome-wide level of homozygosity was almost three times higher in Holiday (33%) than in Korona (13%). This overall predominance of Holiday was also reflected in 14 linkage groups (2A-D, 3A, 3B, 3D, 4B, 4C, 5D, 6C, 6D, 7A and 7D) (Figure 5). However, one linkage group showed higher homozygosity for Korona (LG 5C). Additionally, 8 linkage groups were (nearly) completely heterozygous for both parents (1A, 1B, 3C, 4A, 4D, 5A, 5C and 7B). The overlap of homozygous regions between Holiday and Korona was 125 cM, which is close to the expected 88 cM. For Holiday, the observed level of homozygosity was similar to the theoretically expected 29% based on pedigree kinship coefficients, whereas for Korona, the observed level was more than three times higher than the expected 3.6%.

### Haplotype sharing (kinship)

Holiday and Korona share four independent common ancestors, Aberdeen, Ettersburg 450, Howard 17 and Missionary (Figure 1), which are expected to contribute up to 49% and 21% of the Holiday and Korona genomes, respectively. This level of relatedness makes is likely that Holiday and Korona share marker haplotypes that are identical by descent. The pedigree-derived kinship coefficient between Holiday and Korona was calculated as 0.06 (Table 2). This level of relatedness means that when we pick an allele at a locus in Holiday and then do the same for Korona, the chance that the two alleles are identical by descent is 6%. The actual kinship coefficient estimated from the linkage map was 2.5 times higher at 0.16 (Figure 5). Linkage groups in which both parents were homozygous generally also contained high kinship coefficients (e.g., 1D, 6A, 6B and 7A). A clear exception was homoeologue 7C in which no kinship was found even though this LG had very high levels of homozygosity for both parents. Conversely, on homoeologue 7D we found little shared homozygosity but a very high level of kinship for the heterozygous regions. Homoeologues 2C, 3B, 3C, 4A-C, 5B and 7C had very low kinship coefficients, indicating a high level of diversity between the cultivars.

**Table 2:** Pedigree based kinship coefficients for Holiday and Korona and their common ancestors

**Variety**	**Holiday**	**Korona**
Aberdeen	0.112	0.086
Ettersburg 450	0.071	0.036
Missionary	0.163	0.015
Howard 17	0.140	0.076
Holiday	0.645	0.060
Korona	0.060	0.518

The inbreeding values of Holiday and Korona can be calculated from this table using the kinship coefficient with self = 0.5* (1 + inbreeding value). This analysis amounts to inbreeding values of 0.29 and 0.036 for Holiday and Korona, respectively.

### Duplicated microsatellites

A total of 19 SSR primer pairs yielded amplicons that mapped to more than one heterologous chromosome (Table 3). Of these primer pairs, six had previously been found to be multi-locus SSRs (CFVCT023, CFVCT032, EMFn181, EMFv104, EXP1A and Fvi6b [6,15-18]). For five primer pairs (BFACT048, CFVCT005, EMFv104, UDF033 and UDF056), we found that at least one of the primers was present in regions for which the LTRharvest algorithm found putative retro-transposons in the diploid reference genome (Table 3). For six primer pairs (BFACT041, BFACT048, CFVCT008, EMFv142, EXP1A and Fvi6b), the flanking sequence was located within a genic region that, at least in other species, had high homology to the sequences of large gene families.

**Table 3:** List of duplicated microsatellites in the octoploid Holiday x Korona map

**Marker**	**HxK nr of distinct alleles**	**Mapped LGs**	**Pseudomolecules_v1.1_sign_hits**	**Transposable element (LTRharvest)**	**Inside gene sequence**	**Large gene family**	**Similarity of best_scaffold 4 k region**
BFACT041	21	1,4	0,1,2,3,4,5,6,7	n	y	y	Fragaria x ananassa beta-1,3-glucanase (BG2-2) gene
BFACT048	17	1,2,3,4,5,7	2,3,4,5,6,7	y	y	y	Within intron of populus trichocarpa cytochrome P450 (CYP721)
CFVCT005	12	1,4,6	0,1,2,3,4,7	y	n	n	Not within region with apparant function
CFVCT008	6	4,5,6,7	0,1,2,3,4,5,6,7	n	y	y	Fragaria x ananassa beta-1,3-glucanase (BG2-2) gene, complete cds
CFVCT018	24	1,2,7	0,1,2,3,4,5,6,7	n	n	n	Not within region with apparant function
CFVCT023	12	5,7	7	n	n	n	Not within region with apparant function
CFVCT032	12	3,4,7	0,1,2,3,4,5,6	n	n	n	Not within region with apparant function
EMFn181	33	1,3,4,5,6,7	3,4,5,6	n	n	n	Not within region with apparant function
EMFn198	12	1,3,4,6	no hits				no scaffold
EMFn225	8	5,6	0,1,2,3,4,5,6,7	n	n	n	Not within region with apparant function
EMFn230	4	1,6	no hits				no scaffold
EMFv019	7	1,3	1	n	n	n	Not within region with apparant function
EMFv104	11	3,6	3,6	y	y	n	Within intron of ferredoxin-dependent glutamate synthase
EMFv142	10	2,4	3,4,7	n	y	y	Within intron of adiponectin receptor protein 1-like (LOC100777701), mRNA
EXP1A	14	4,7	3,5,6,7	n	y	y	Expansin (EXP2)
Fvi6B	14	3,6	6	n	y	y	Within intron of chromatin remodeling complex subunit (CHR923)
UDF004	7	3,5	3	n	y	n	Within intron of molybdopterin cofactor sulfurase (ABA3)
UDF033	14	1,2,3	3,4,7	y	y	n	Pyrus pyrifolia genes for F-box proteins and S2-RNase, complete cds, haplotype: S2
UDF056	5	2,3	4,6	y	n	n	Not within region with apparant function

Finally, for nine primer pairs (CFVCT018, CFVCT023, CFVCT032, EMFn181, EMFn198, EMFn225, EMFn230, EMFv019 and UDF004), we could not find any putative explanation for their targeting of heterologous loci. Two of these (EMFn198 and EMFn230) did not yield sufficiently specific hits in the reference genome to do further analysis. Another two (EMFn181 and EMFn225) corresponded to loci of varying positions among the four homoeologues of a chromosome and finally, two pairs (CFVCT018 and EMFn198) corresponded to loci of varying position within a homoeologue. This result strongly indicates that markers EMFn181, EMFn225 CFVCT018 and EMFn198 represent mobile elements, which is consistent with the lack of adjacent markers showing similar behaviour.

### Comparison to the diploid genome

**Figure 6:**
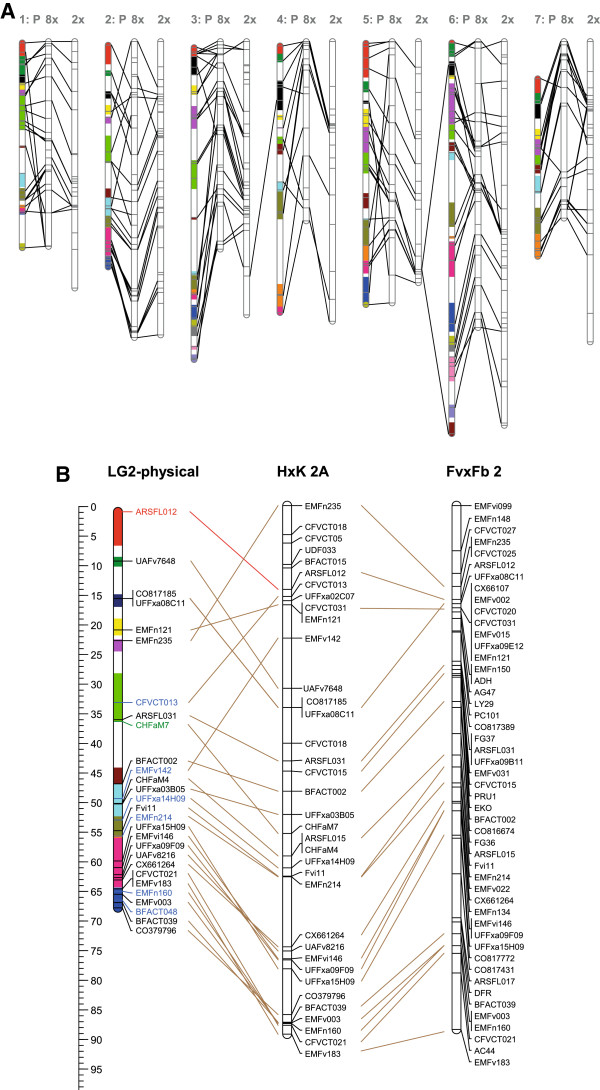
**Comparative mapping octoploid vs diploid. A**: An overview of comparative mapping between the physical reference genome, a representative octoploid homoeologue, and the diploid Fv xFb map [51]. Coloured bar segments represent scaffolds. **B**: A more detailed figure of LG2 including marker names and genetic positions. The ruler represents the position in cM for genetic maps, and the position in mega-bases for the physical map. The latter are multiplied by three in order to better fit the scale of the genetic maps. Blue font indicates a marker for which only 1 primer hit, but with 100% identity. Red font indicates a marker for which only 1 primer hit and the identity was not 100%. The lines for order comparison were drawn from locus name to position instead of position to position in order to facilitate traceability of locus names. The details of other LGs are presented in Additional file 3: Figure S2.

For a comparison of marker order between the pseudo-chromosomes of the diploid *F. vesca* reference genome (V 1.1) [13,50], the most representative homoeologues of our octoploid map and the diploid FvxFb map [51] are presented in Figure 6 and Additional file 3: Figure S2. The overall marker order conservation between the diploid physical and octoploid genetic map was found to be high, but nevertheless, it showed some discrepancies, which were classified into two types. Type I involved inversions in marker order over relatively small (scaffold size) distances. Two clear examples occur at the distal end of LG2 where the orientation of scaffolds seems to be inverted (Figure 6). The type II discrepancy involved mostly single loci that showed large differences in their position and order from the physical map to the octoploid genetic map. Examples include the marker loci EMFn235, EMFn121 and UFFxa08C11 for LG2 (Figure 6, Additional file 3: Figure S2). Overall, our genetic map and the diploid FvxFb genetic map were consistent with each other, especially in the case of type II discrepancies. This could indicate that there are still some mistakes in the orientation and position of a number of scaffolds in the diploid physical pseudo-chromosome maps. Our map of the octoploid strawberry may thus help to further improve the physical map.

## Discussion

In this study, we used the MADCE method [27] to develop an integrated genetic map of two octoploid strawberry cultivars that, for the first time for a polyploid plant species, included comprehensive haplotype information, even for homozygous regions and areas with null alleles. The benefits of having such extensive haplotype information are discussed in the following sections.

### Map length and marker density

The map length of 2050 cM (corrected for homozygosity) is largely in line with the results of previous studies [6,15-17,22]. The marker density of one marker per every 3.6 cM does not provide improvements over some previously reported linkage maps in octoploid strawberry [16,22]. However, the use of MADCE allowed us to maximise the number of segregating loci per primer pair, allowing for better comparisons of marker order retention between homoeologues. In addition, the very precise pinpointing of homozygous regions showed that marker saturation in these areas for obtaining a better resolution would be futile, as was also indicated previously by Sargent et al. [16].

### Denotation of sub-genomes

No convention exists regarding the differentiation and notation of sub-genomes. Here, we distinguished the sub-genomes based on their sequence divergence from *F. vesca* using molecular markers. This approach is in contrast to those of previous studies where technical features such as number of loci and map length were used to distinguish the homoeologues [6,14-22]. These parameters are affected by the level of homozygosity and may thus largely vary with the parents that were used for crossing. We believe that our approach, though rudimentary, is biologically more relevant and that in the near future the link between the octoploid sub-genomes and their diploid and tetraploid ancestors will become better specified, as has occurred for bread wheat [23].

Because strawberry is fully diploidized and for the ease of distinction, we denoted the sub-genomes with the letters A–D. However, if in the near future octoploid strawberries are shown to have originated from two tetraploid species of different origins, as suggested by Rousseau-Gueutin et al. [56], and as supported by our data on LGs 1, 5 and 7, then we may adopt an identical homoeologue naming convention.

Currently, the ‘Holiday’ x ‘Korona’ map is used for the mapping of thousands of SNP markers from the recently released Axiom^®^ Strawberry Genotyping Array (also called International Strawberry 90 K SNP array or IStraw90), which was generated in a joint effort of the USDA-SCRI RosBREED project (http://www.rosbreed.org) and Affymetrix Ltd (Santa Clara, CA, USA). Now that large scale SNP arrays have come into use for strawberry, the use of a similar naming convention for octoploid maps should become straightforward due to the expected high number of common markers.

### Genomic organization of homoeologues: collinearity & re-arrangements

#### Homoeologue collinearity and re-arrangements

The marker order between homoeologous linkage groups was highly collinear, as observed in previous studies [6,15-17,22]. Inconsistencies spanning very small distances are more likely to be attributable to scoring errors, missing values, the use of markers that are not equally informative, and differences in recombination rates between the single parent maps than actual genomic rearrangements.

We described for the first time a large inversion in a linkage map of octoploid strawberry. This inversion on LG2D spanned almost 30 cM and was verified in an independent mapping population. Because we could not trace this inversion in the linkage maps of *Fragaria vesca* and *Fragaria bucharica*[10,11,42,51,57], it should derive from one of the other ancestors of *Fragaria x ananassa,* or, less likely, may have occurred after polyploidisation. Apart from the LG2D inversion, we also found evidence that LG2C may contain a rearrangement, although the evidence was less clear. It would be interesting to further investigate these rearrangements using different progenies, higher marker densities and fluorescent in-situ hybridisation (FISH, similar to Tang et al. [58]), as the rearrangements could reveal interesting insights into the relationship between the octoploid strawberry and its diploid relatives.

### Breeding signatures in mapping parents

#### Homozygosity

The information on homozygosity and haplotype sharing generated by our genetic map revealed interesting features for both of the parental varieties, which allows us to hypothesise the possibility of breeding signatures. On a genome-wide level, the homozygosity in Holiday was found to be similar to what was theoretically expected (approximately 30%), whereas for Korona, the inbreeding level was much higher than expected (13% vs. 3.6%). Holiday has a history of heavy inbreeding, and Korona does not. It is likely that normalisation occurs for homozygosity, where extreme levels of homozygosity and extreme levels of heterozygosity are not favoured during selection. This trend may be especially true for high levels of homozygosity, as such levels are known to lead to inbreeding depression in strawberry [59,60]. In crop species, certain traits of high agronomic value, such as plant size, adaptability and vigour, frequently favour heterozygous states [61]. Conversely, traits such as fruit firmness, shape and size are often inherited recessively or occur due to the additive nature of alleles and thereby favour homozygous states [62]. The differences in phenotype between the parents corroborate this hypothesis. The heavily inbred cultivar Holiday was purposefully bred for high fruit firmness and skin toughness, whereas Korona is a popular garden variety because of its adaptability and taste. However, it suffers from soft fruits and irregular fruit shape. Both varieties exhibit large fruit size and yield. There is a high probability that the differences and similarities in the distribution of homozygosity along the genome reflect the phenotype differences and similarities of the parental lines. Genes controlling fruit firmness, shape and skin vulnerability may therefore be located in areas where Holiday is homozygous and Korona is heterozygous, whereas genes controlling traits that favour agronomic performance are more likely to be located in regions where both parents are heterozygous.

#### IBD of haplotypes (kinship)

For Holiday and Korona, linkage map-derived kinship is more than twice as high as expected from their pedigrees. This result could indicate positive selection of these shared genetic regions. The two varieties with the theoretically largest contribution to the kinship between Holiday and Korona are Howard 17 and Aberdeen. These two varieties have been used extensively as parents in early 20th century strawberry breeding [63] and are therefore present in the pedigrees of many modern varieties. It is very likely that certain genomic regions of these founders are under positive selection, which would result in a higher than expected level of kinship in their descendants. Another explanation for the relatively high level of kinship could be the presence of close common ancestry among the founders of Holiday and Korona. The distribution of the shared haplotypes appears to be non-random. Certain chromosomes, such as 7A and 7D, were found to have almost three times the average haplotype-sharing, whereas all homoeologues of chromosome 4 had virtually no shared haplotypes. This result could be a coincidence but may also be due to positive or purifying selection for specific regions. Tracing the shared haplotypes between Holiday and Korona over a pedigree to their founders as well as their descendants could further clarify which haplotypes are under strong positive or purifying selection. These could be interesting for marker assisted breeding, even without knowing the associated trait(s).

### Duplications

Nineteen (10%) of the 186 microsatellite markers tested mapped to two or more heterologous chromosomes. Six of these had previously been reported as duplicated [6,15,16], and some were suggested to be remnants of a putative ancient chromosomal duplication event [16]. Our findings did not support the presence of ancient duplication events, as we could not find a clear pattern where the same heterologous chromosome segments were consistently being amplified by multiple duplicated microsatellites. The occurrence of several of these markers in known transposable elements and large gene families provides further evidence against the hypothesis of duplication events.

### Comparison to the diploid *Fragaria* genome

The comparative mapping revealed a generally high level of collinearity between the octoploid genetic map and the diploid physical and genetic map. This result is in line with that of previous studies [6,15-17,22]. However, we did find some discrepancies, which in most cases showed that the octoploid genetic map had better collinearity with the diploid Fv × Fb genetic map [51] than with the diploid *Fragaria vesca* pseudo chromosomes (v1.1). This result indicates that most of the divergence between our genetic maps and the physical map are due to limitations of the physical map. A possible explanation for this result could be that some of the scaffolds have been mapped and oriented with a BIN set comprised of a relatively low number of individuals. Knowledge of the identity of erroneously placed or erroneously oriented scaffolds as provided by this study may be of great help when fine-mapping genes of interest. However, it is not always possible to pinpoint which of the discrepancies are due to errors in the genetic maps or pseudo-chromosomes or due to real rearrangements. We could therefore not positively confirm nor reject the rearrangements observed by Sargent et al. [16] on LGs 1, 3 and 4.

## Conclusions

The MADCE approach enabled the full assessment of marker haplotypes for sets of SSR-loci across the four homoeologues. It also enabled the identification of genomic rearrangements, and the discernment of homoeologues based on their similarity to the *F. vesca* genome. Moreover, we were able to assess the level and distribution of homozygosity and haplotype-sharing, which could indicate breeding signatures. The availability of haplotype information is crucial to go from mapping population-derived QTLs to marker-assisted selection in breeding germplasm. Haplotype information will also prove to be a valuable tool in several other aspects of strawberry breeding, such as parent selection, the verification of pedigree information and IBD analysis. New technologies such as SNP arrays and Genotyping by Sequencing (GBS) could speed up the availability of such information in allopolyploids, if coupled with appropriate methodologies to discern the different sub-genomes. We hope that with this study we have provided a significant step towards the availability of such comprehensive genetic information in strawberry.

## Competing interests

The authors declare that they have no competing interests.

## Authors’ contributions

TvD Performed experimental work, participated in the design of this study, performed linkage mapping and data analysis and drafted the manuscript. GP performed experimental work and the comparative mapping. AP performed experimental work and data analysis. YN performed experimental work, HYT performed experimental work and data analysis, BM was responsible for the plant material and participated in design of study, RV participated in coordination and helped draft the manuscript. EvdW conceived the study, participated in its design and coordination and helped to draft the manuscript. All authors read and approved the final manuscript.

## Supplementary Material

Additional file 1: Table S1 Primer pairs. Excel sheet with primer pairs used in this study. Click here for file

Additional file 2: Figure S1 Holiday x Korona All Maps. All linkage maps of this study. For further description see Figure 2 of manuscript. Click here for file

Additional file 3: Figure S2 Comparative maps All Chromosomes. Comparison between our octoploid linkage maps and the diploid physical and genetic maps. For further description see Figure 6 of manuscript. Click here for file
