# Supplementary figures and images for: Genomic rearrangements and signatures of breeding in the allo-octoploid strawberry as revealed through an allele dose based SSR linkage map
Source: BMC Plant Biol. 2014 Mar 1;14:55. doi: 10.1186/1471-2229-14-55 (PMC3944823; doi:10.1186/1471-2229-14-55)

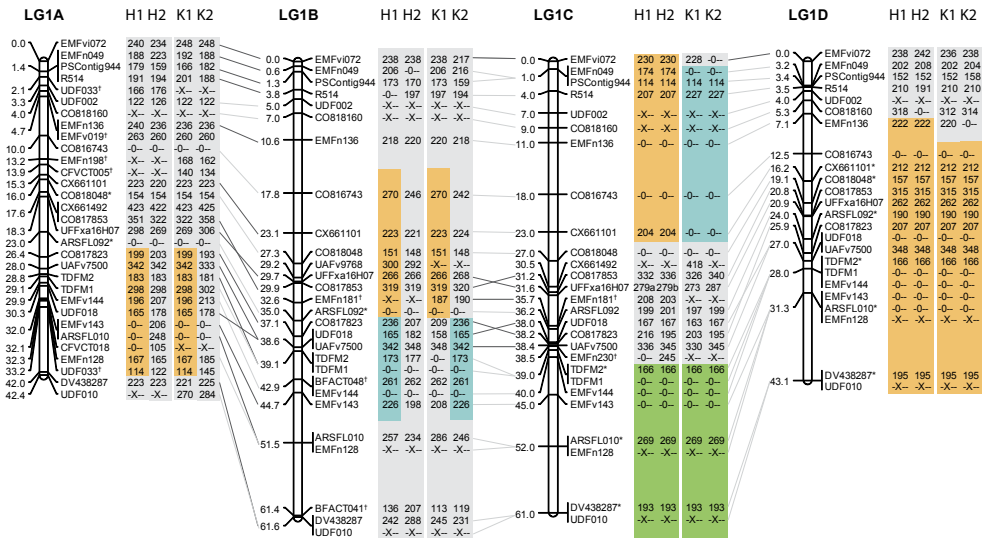

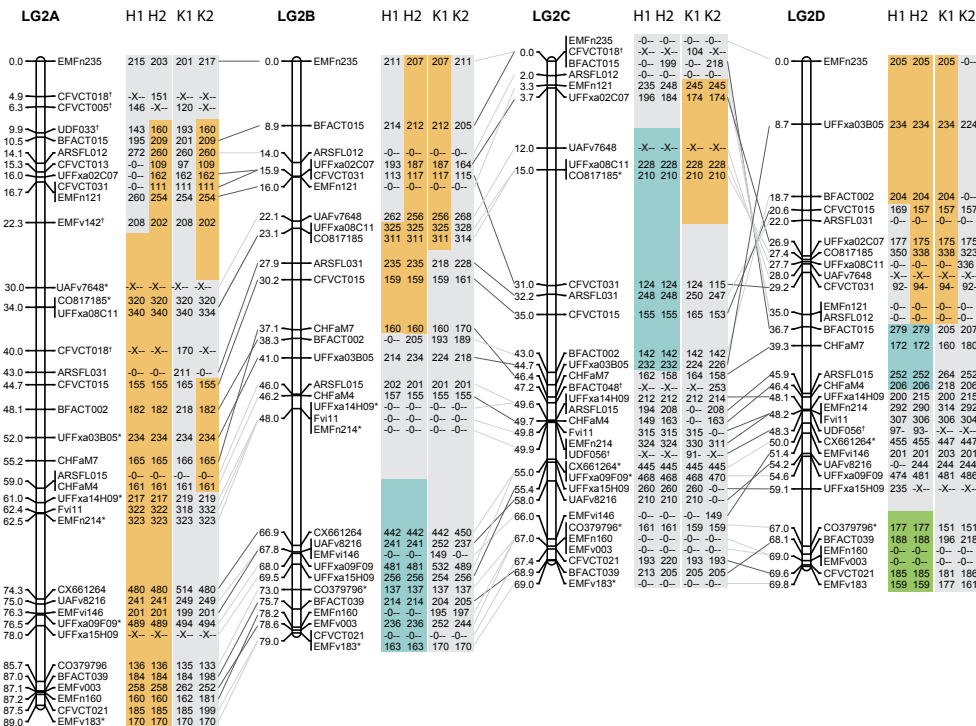

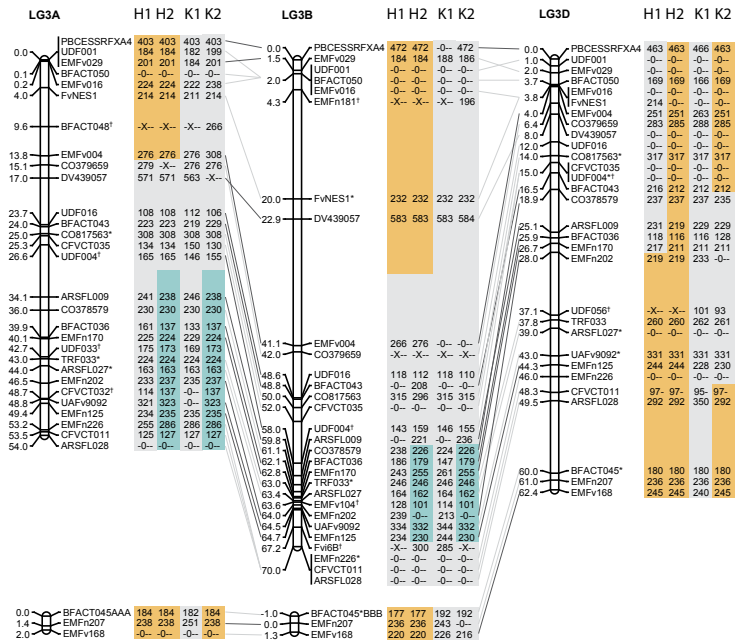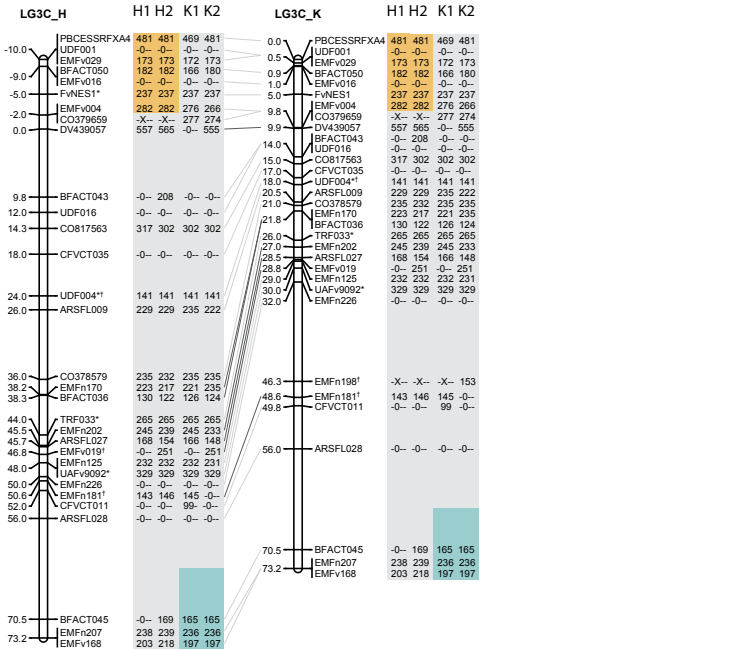

## LG4A

## LG4B

## LG4C

## LG4D

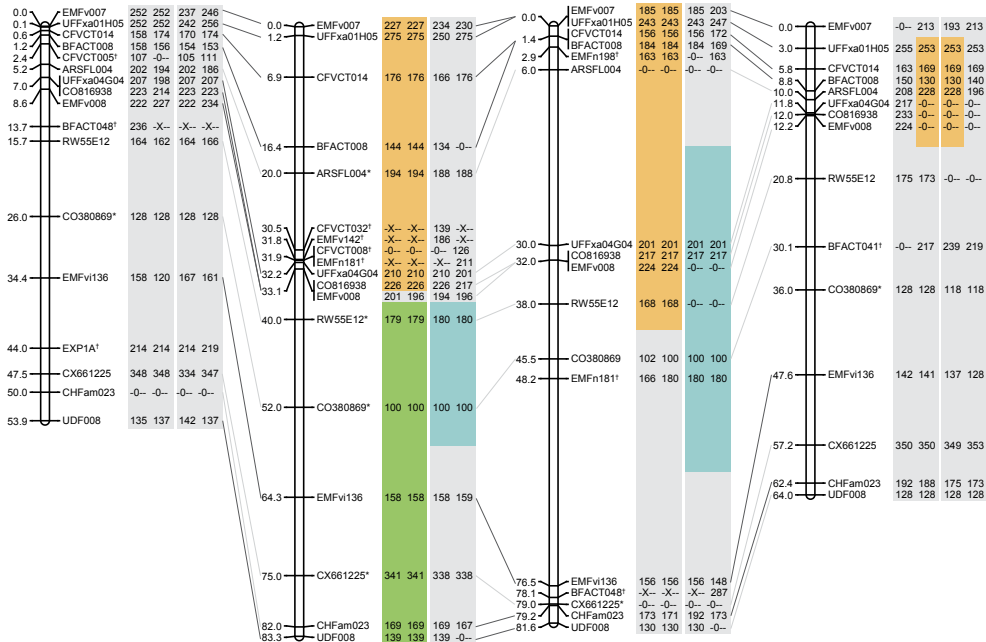

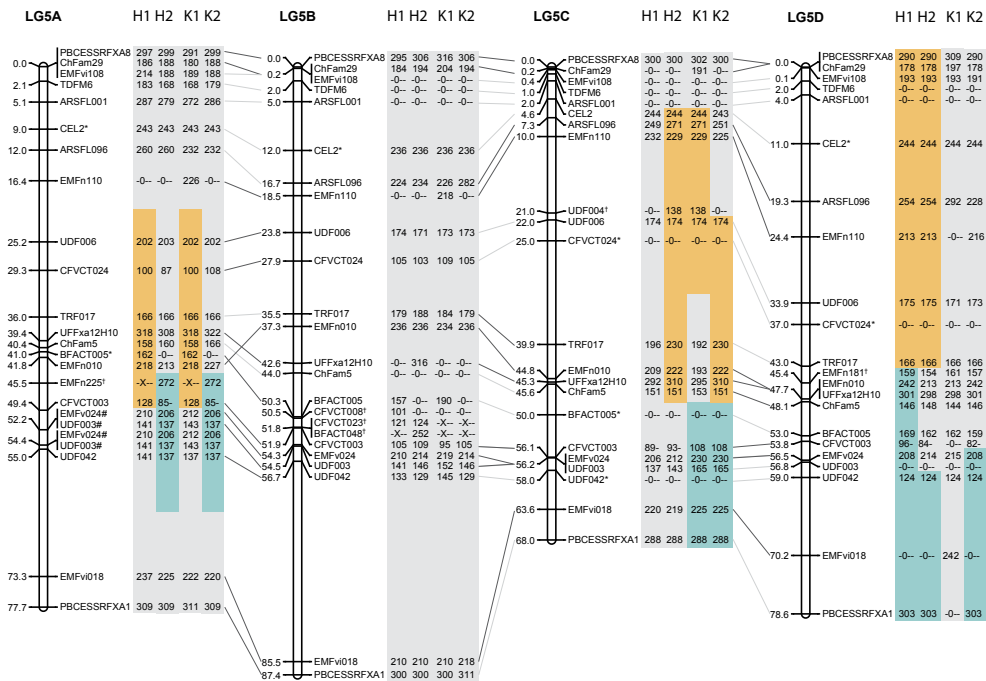

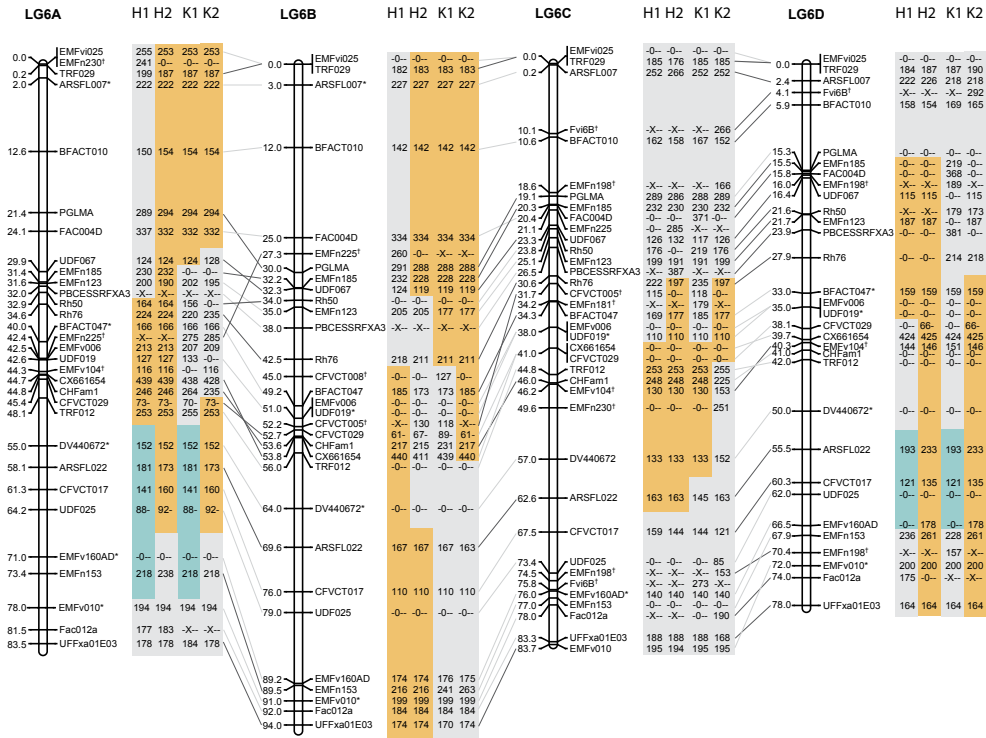

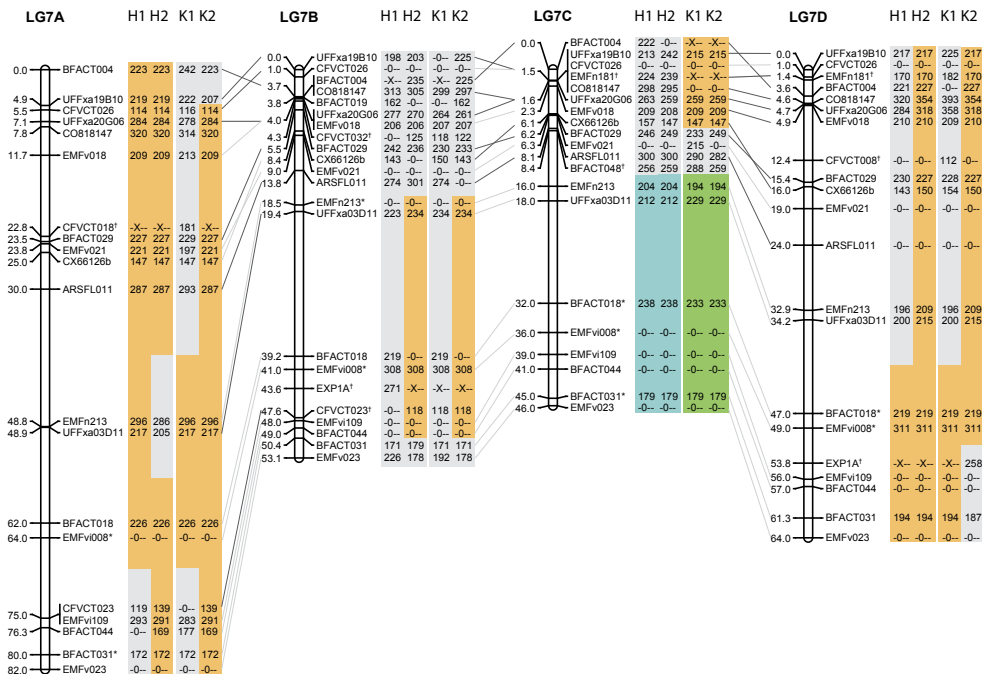

Supplement: Additional file 2: Figure S1 — Holiday x Korona All Maps. All linkage maps of this study. For further description see Figure 2 of manuscript. [file 1471-2229-14-55-S2.pdf]
