# Supplementary material for: Genomic rearrangements and signatures of breeding in the allo-octoploid strawberry as revealed through an allele dose based SSR linkage map
Source: BMC Plant Biol. 2014 Mar 1;14:55. doi: 10.1186/1471-2229-14-55 (PMC3944823; doi:10.1186/1471-2229-14-55)
Supplement: Additional file 3: Figure S2 — Comparative maps All Chromosomes. Comparison between our octoploid linkage maps and the diploid physical and genetic maps. For further description see Figure 6 of manuscript. [file 1471-2229-14-55-S3.pdf]

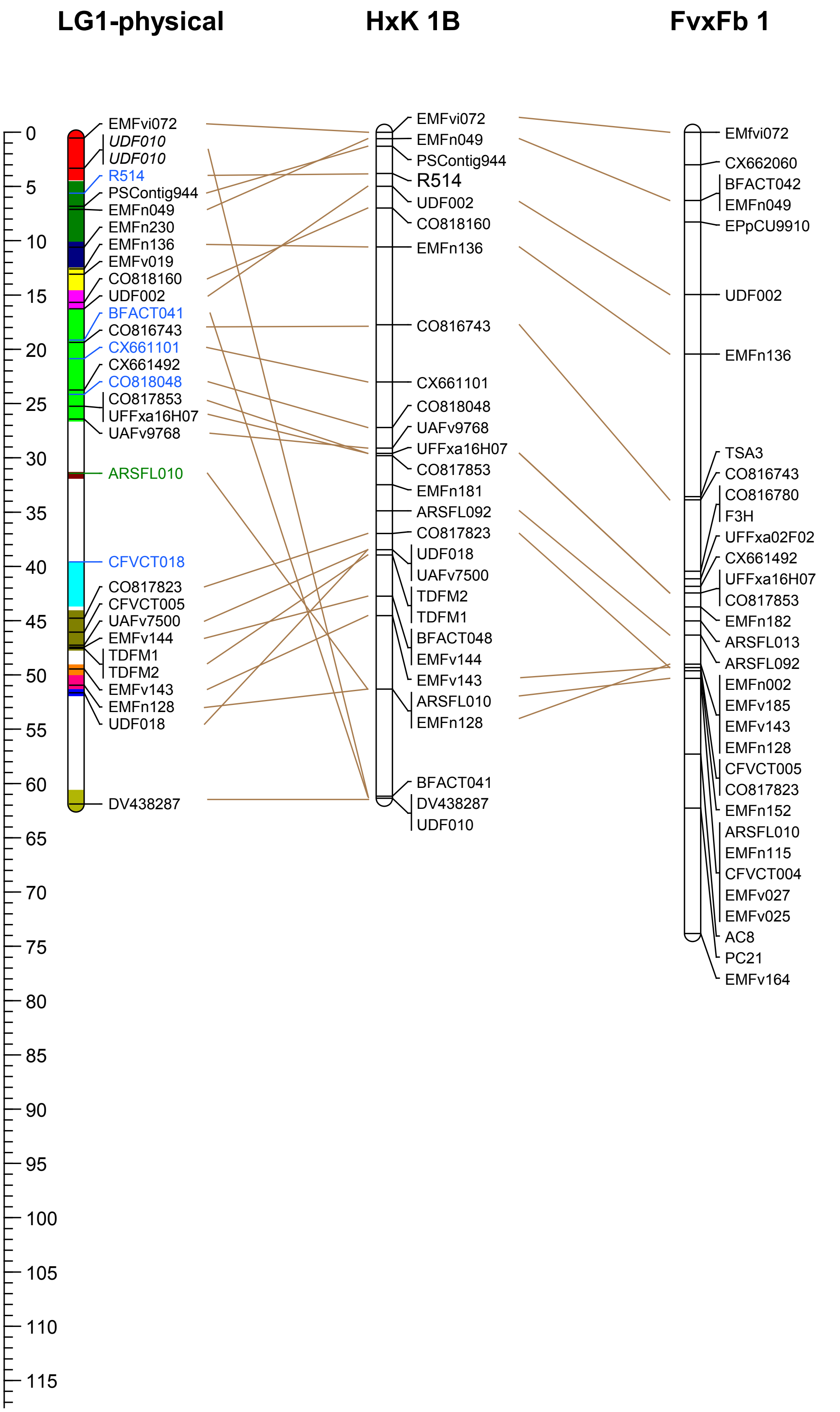

LG1-physical
HxK 1B
FvxFb 1
0
5
10
15
20
25
30
35
40
45
50
55
60
65
70
75
80
85
90
95
100
105
110
115
EMFvi072
UDF010
UDF010
R514
PSContig944
EMFn049
EMFn230
EMFn136
EMFv019
CO818160
UDF002
BFACT041
CO816743
CX661101
CX661492
CO818048
CO817853
UFFxa16H07
UAFv9768
ARSFL010
CFVCT018
CO817823
CFVCT005
UAFv7500
EMFv144
TDFM1
TDFM2
EMFv143
EMFn128
UDF018
DV438287
EMFvi072
EMFn049
PSContig944
R514
UDF002
CO818160
EMFn136
CO816743
CX661101
CO818048
UAFv9768
UFFxa16H07
CO817853
EMFn181
ARSFL092
CO817823
UDF018
UAFv7500
TDFM2
TDFM1
BFACT048
EMFv144
EMFv143
ARSFL010
EMFn128
BFACT041
DV438287
UDF010
EMfvi072
CX662060
BFACT042
EMFn049
EPpCU9910
UDF002
EMFn136
TSA3
CO816743
CO816780
F3H
UFFxa02F02
CX661492
UFFxa16H07
CO817853
EMFn182
ARSFL013
ARSFL092
EMFn002
EMFv185
EMFv143
EMFn128
CFVCT005
CO817823
EMFn152
ARSFL010
EMFn115
CFVCT004
EMFv027
EMFv025
AC8
PC21
EMFv164

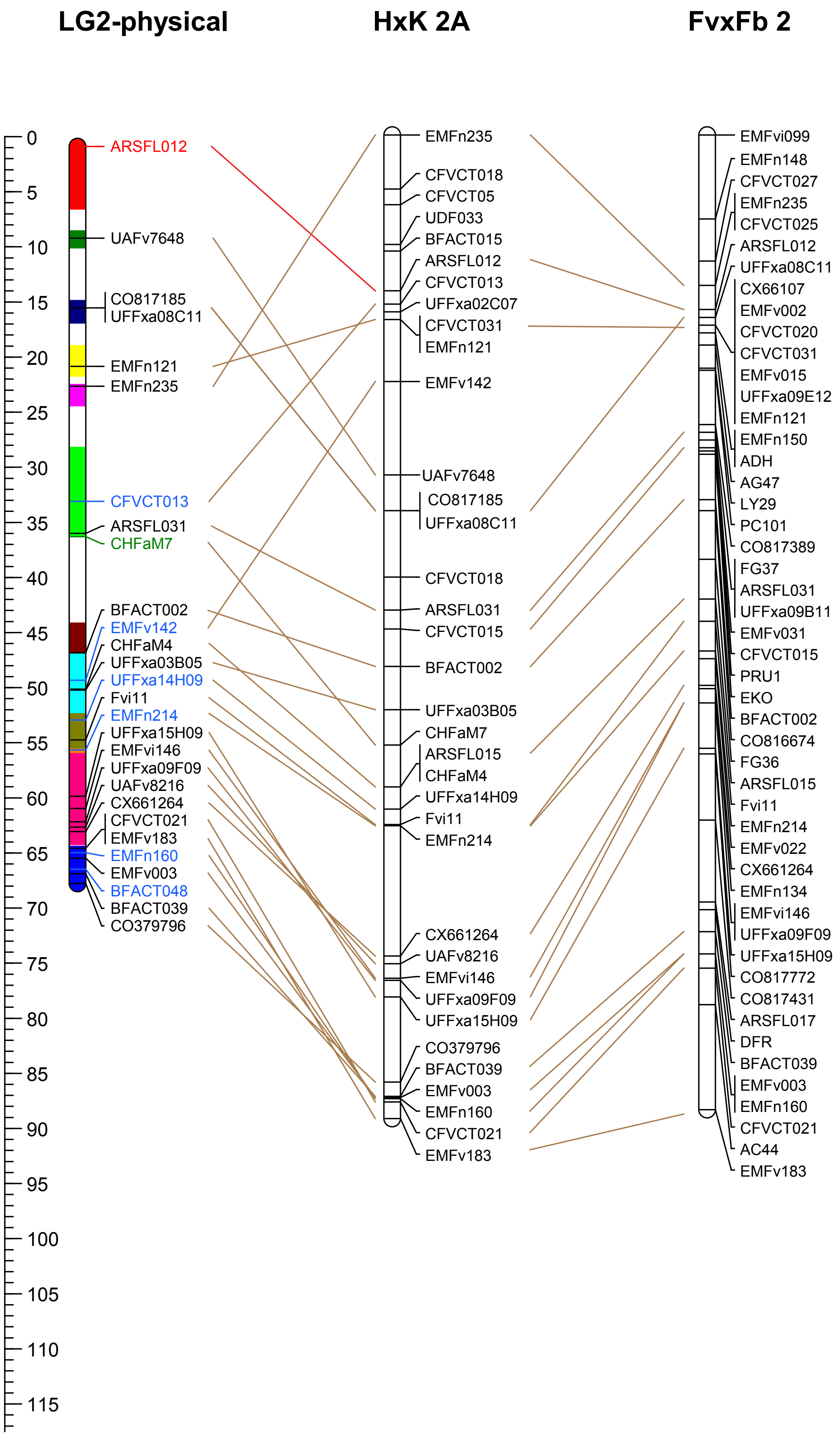

LG2-physical
HxK 2A
FvxFb 2
0
5
10
15
20
25
30
35
40
45
50
55
60
65
70
75
80
85
90
95
100
105
110
115
ARSFL012
UAFv7648
CO817185
UFFxa08C11
EMFn121
EMFn235
CFVCT013
ARSFL031
CHFaM7
BFACT002
EMFv142
CHFaM4
UFFxa03B05
UFFxa14H09
Fvi11
EMFn214
UFFxa15H09
EMFvi146
UFFxa09F09
UAFv8216
CX661264
CFVCT021
EMFv183
EMFn160
EMFv003
BFACT048
BFACT039
CO379796
EMFn235
CFVCT018
CFVCT05
UDF033
BFACT015
ARSFL012
CFVCT013
UFFxa02C07
CFVCT031
EMFn121
EMFv142
UAFv7648
CO817185
UFFxa08C11
CFVCT018
ARSFL031
CFVCT015
BFACT002
UFFxa03B05
CHFaM7
ARSFL015
CHFaM4
UFFxa14H09
Fvi11
EMFn214
CX661264
UAFv8216
EMFvi146
UFFxa09F09
UFFxa15H09
CO379796
BFACT039
EMFv003
EMFn160
CFVCT021
EMFv183
EMFvi099
EMFn148
CFVCT027
EMFn235
CFVCT025
ARSFL012
UFFxa08C11
CX66107
EMFv002
CFVCT020
CFVCT031
EMFv015
UFFxa09E12
EMFn121
EMFn150
ADH
AG47
LY29
PC101
CO817389
FG37
ARSFL031
UFFxa09B11
EMFv031
CFVCT015
PRU1
EKO
BFACT002
CO816674
FG36
ARSFL015
Fvi11
EMFn214
EMFv022
CX661264
EMFn134
EMFvi146
UFFxa09F09
UFFxa15H09
CO817772
CO817431
ARSFL017
DFR
BFACT039
EMFv003
EMFn160
CFVCT021
AC44
EMFv183

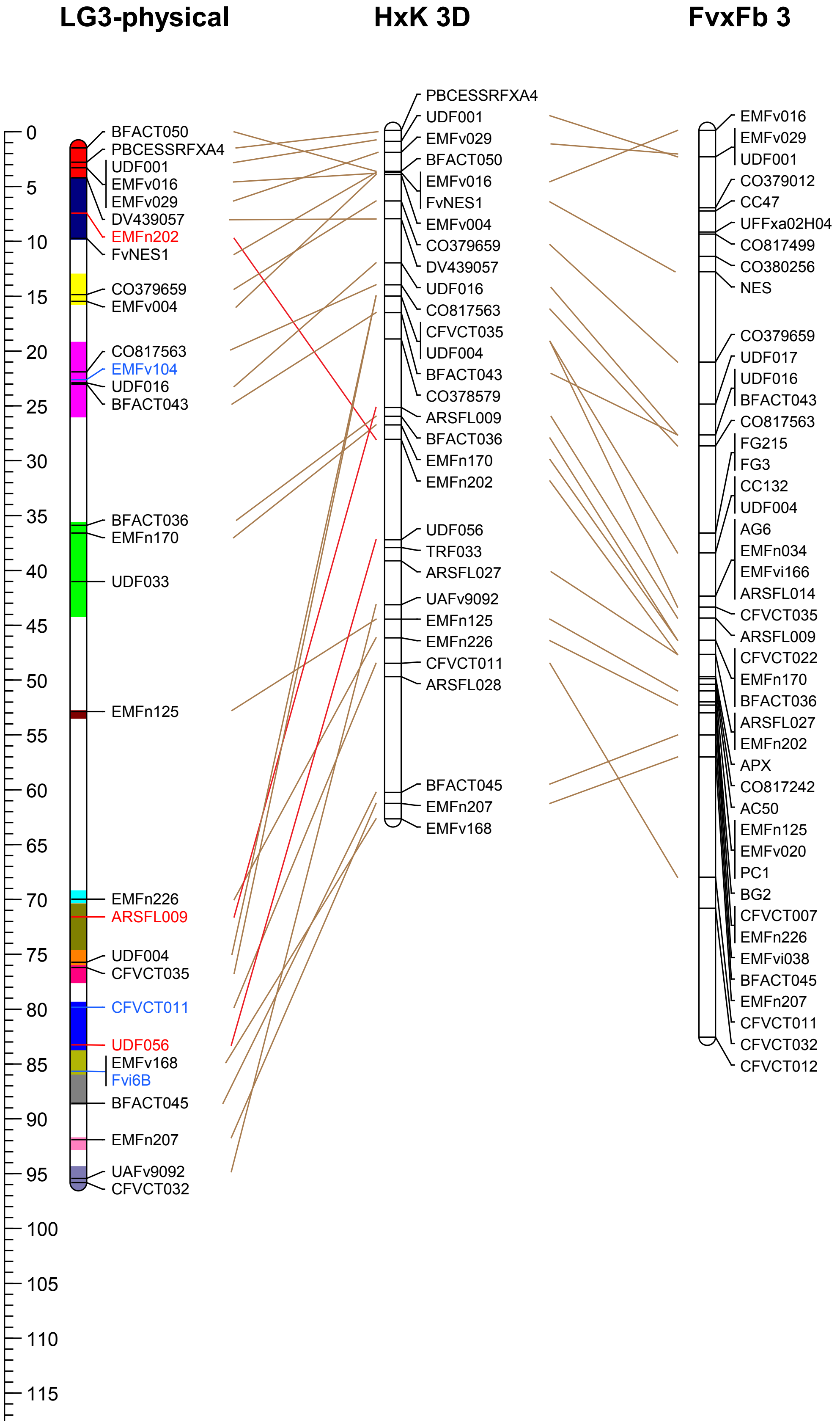
LG3-physical
HxK 3D
FvxFb 3
BFACT050
PBCESSRFXA4
UDF001
EMFv016
EMFv029
DV439057
EMFn202
FvNES1
CO379659
EMFv004
CO817563
EMFv104
UDF016
BFACT043
BFACT036
EMFn170
UDF033
EMFn125
EMFn226
ARSFL009
UDF004
CFVCT035
CFVCT011
UDF056
EMFv168
Fvi6B
BFACT045
EMFn207
UAFv9092
CFVCT032
CO378579
ARSFL009
TRF033
ARSFL027
CFVCT011
ARSFL028
EMFv168
CO379012
CC47
UFFxa02H04
CO817499
CO380256
NES
UDF017
FG215
FG3
CC132
AG6
EMFn034
EMFvi166
ARSFL014
CFVCT022
APX
CO817242
AC50
EMFv020
PC1
BG2
CFVCT007
EMFvi038
CFVCT012

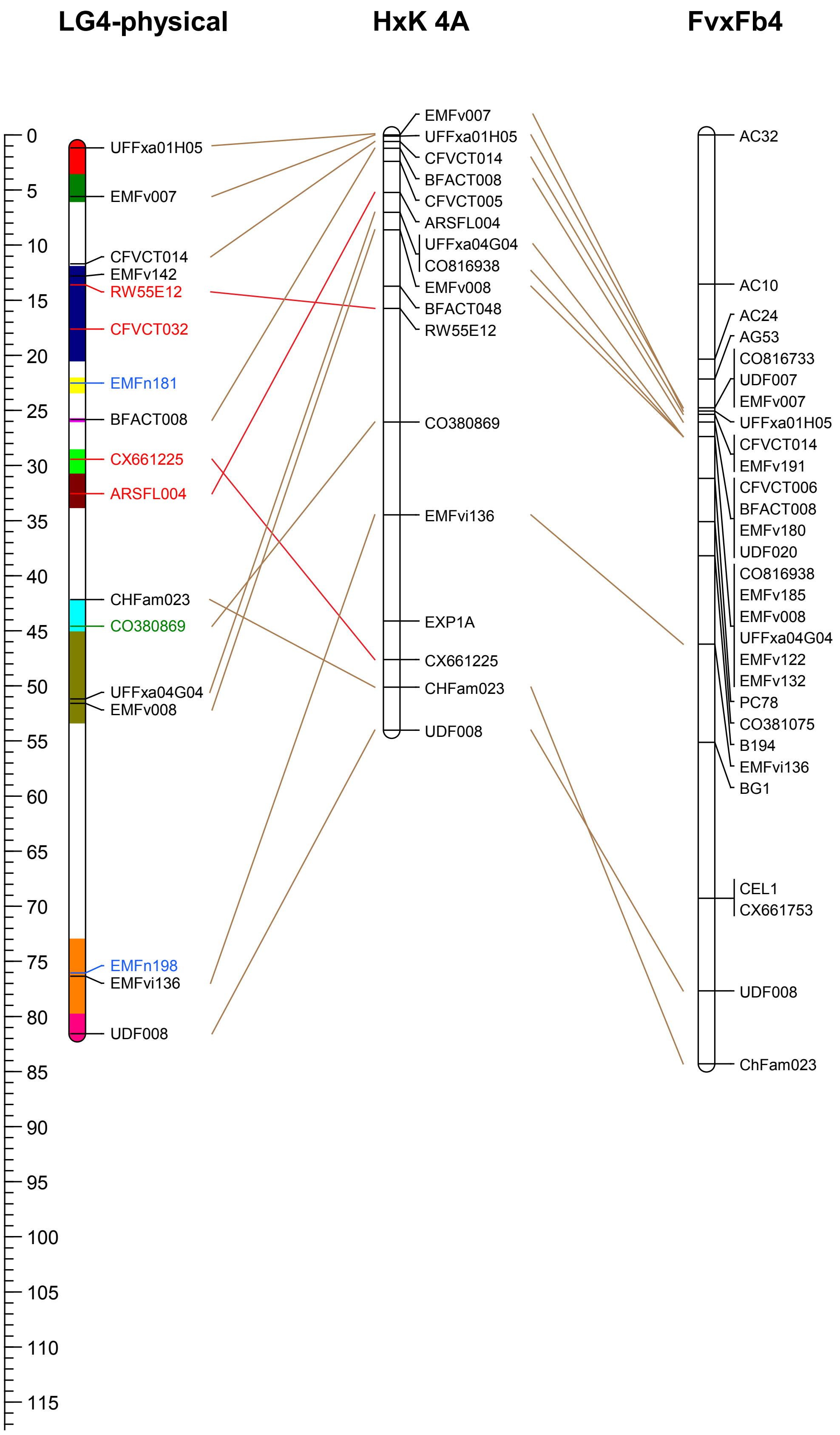
LG4-physical
HxK 4A
FvxFb4
0
5
10
15
20
25
30
35
40
45
50
55
60
65
70
75
80
85
90
95
100
105
110
115
UFFxa01H05
EMFv007
CFVCT014
EMFv142
RW55E12
CFVCT032
EMFn181
BFACT008
CX661225
ARSFL004
CHFam023
CO380869
UFFxa04G04
EMFv008
EMFn198
EMFvi136
UDF008
EMFv007
UFFxa01H05
CFVCT014
BFACT008
CFVCT005
ARSFL004
UFFxa04G04
CO816938
EMFv008
BFACT048
RW55E12
CO380869
EMFvi136
EXP1A
CX661225
CHFam023
UDF008
AC32
AC10
AC24
AG53
CO816733
UDF007
EMFv007
UFFxa01H05
CFVCT014
EMFv191
CFVCT006
BFACT008
EMFv180
UDF020
CO816938
EMFv185
EMFv008
UFFxa04G04
EMFv122
EMFv132
PC78
CO381075
B194
EMFvi136
BG1
CEL1
CX661753
UDF008
ChFam023

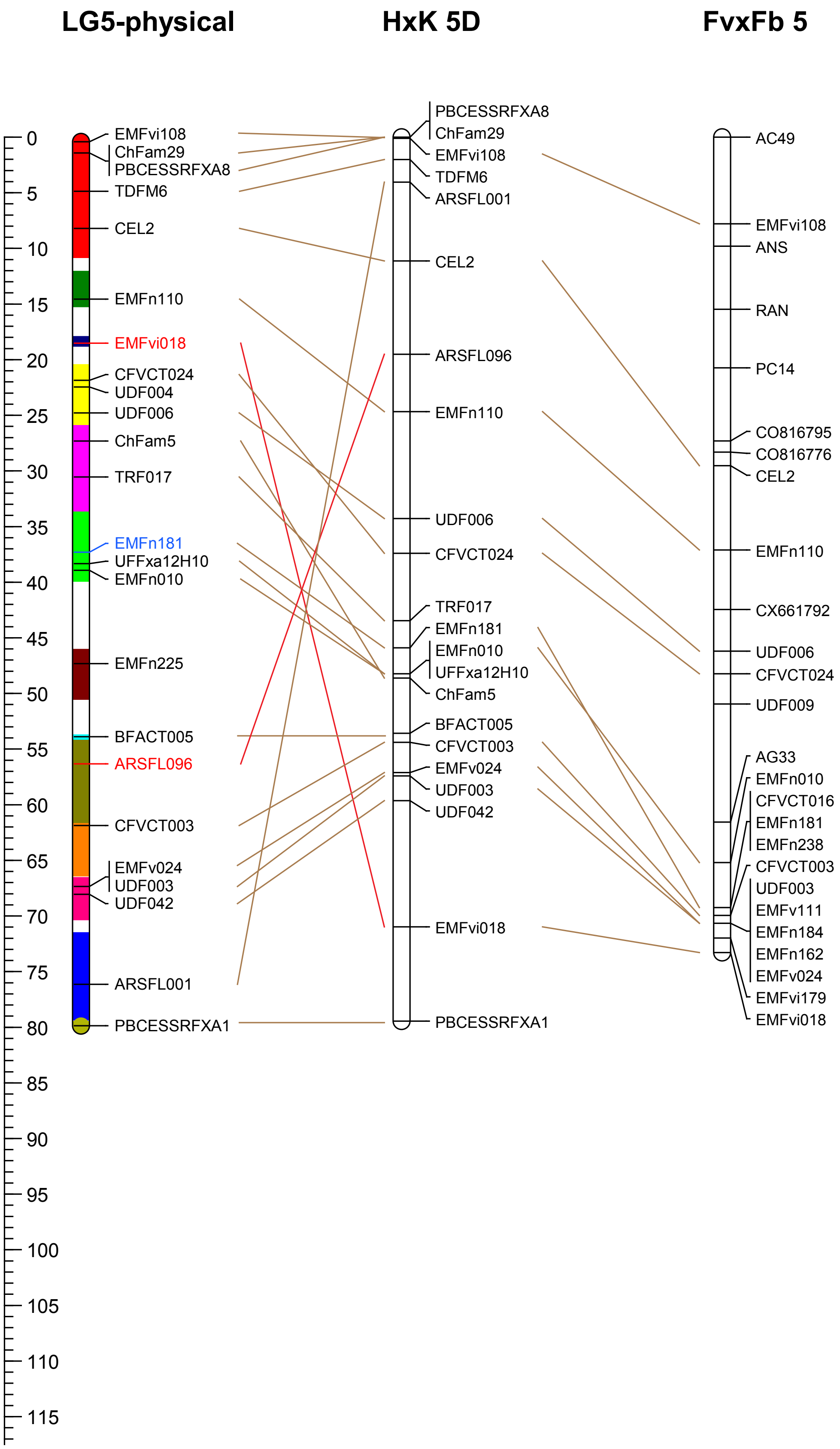

LG5-physical
HxK 5D
FvxFb 5
0
5
10
15
20
25
30
35
40
45
50
55
60
65
70
75
80
85
90
95
100
105
110
115
EMFvi108
ChFam29
PBCESSRFXA8
TDFM6
CEL2
EMFn110
EMFvi018
CFVCT024
UDF004
UDF006
ChFam5
TRF017
EMFn181
UFFxa12H10
EMFn010
EMFn225
BFACT005
ARSFL096
CFVCT003
EMFv024
UDF003
UDF042
ARSFL001
PBCESSRFXA1
PBCESSRFXA8
ChFam29
EMFvi108
TDFM6
ARSFL001
CEL2
ARSFL096
EMFn110
UDF006
CFVCT024
TRF017
EMFn181
EMFn010
UFFxa12H10
ChFam5
BFACT005
CFVCT003
EMFv024
UDF003
UDF042
EMFvi018
PBCESSRFXA1
AC49
EMFvi108
ANS
RAN
PC14
CO816795
CO816776
CEL2
EMFn110
CX661792
UDF006
CFVCT024
UDF009
AG33
EMFn010
CFVCT016
EMFn181
EMFn238
CFVCT003
UDF003
EMFv111
EMFn184
EMFn162
EMFv024
EMFvi179
EMFvi018

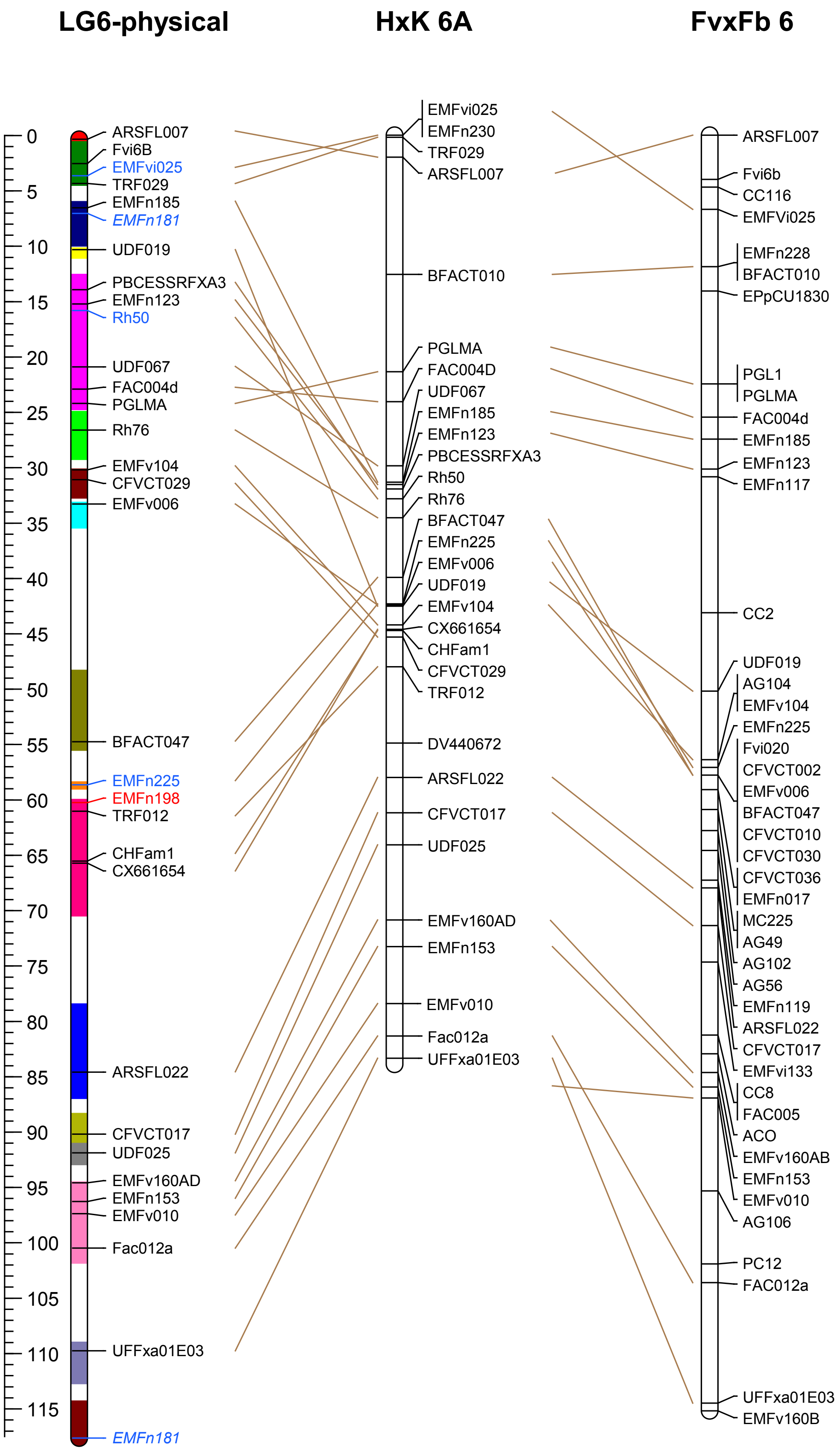
LG6-physical
HxK 6A
FvxFb 6
0
5
10
15
20
25
30
35
40
45
50
55
60
65
70
75
80
85
90
95
100
105
110
115
ARSFL007
Fvi6B
EMFvi025
TRF029
EMFn185
EMFn181
UDF019
PBCESSRFXA3
EMFn123
Rh50
UDF067
FAC004d
PGLMA
Rh76
EMFv104
CFVCT029
EMFv006
BFACT047
EMFn225
EMFn198
TRF012
CHFam1
CX661654
ARSFL022
CFVCT017
UDF025
EMFv160AD
EMFn153
EMFv010
Fac012a
UFFxa01E03
EMFn181
EMFvi025
EMFn230
TRF029
ARSFL007
BFACT010
PGLMA
FAC004D
UDF067
EMFn185
EMFn123
PBCESSRFXA3
Rh50
Rh76
BFACT047
EMFn225
EMFv006
UDF019
EMFv104
CX661654
CHFam1
CFVCT029
TRF012
DV440672
ARSFL022
CFVCT017
UDF025
EMFv160AD
EMFn153
EMFv010
Fac012a
UFFxa01E03
ARSFL007
Fvi6b
CC116
EMFVi025
EMFn228
BFACT010
EPpCU1830
PGL1
PGLMA
FAC004d
EMFn185
EMFn123
EMFn117
CC2
UDF019
AG104
EMFv104
EMFn225
Fvi020
CFVCT002
EMFv006
BFACT047
CFVCT010
CFVCT030
CFVCT036
EMFn017
MC225
AG49
AG102
AG56
EMFn119
ARSFL022
CFVCT017
EMFvi133
CC8
FAC005
ACO
EMFv160AB
EMFn153
EMFv010
AG106
PC12
FAC012a
UFFxa01E03
EMFv160B

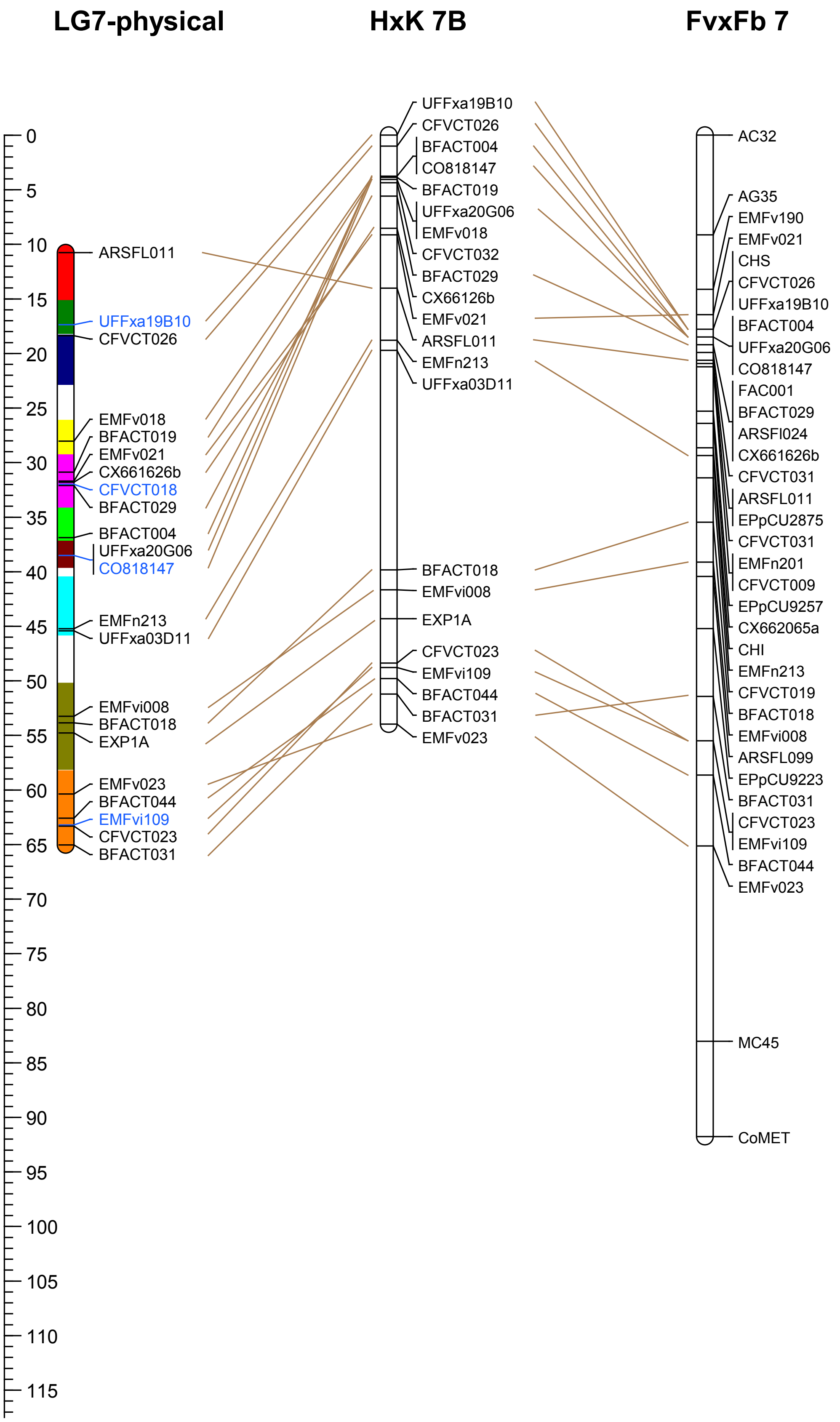
LG7-physical
HxK 7B
FvxFb 7
0
5
10
15
20
25
30
35
40
45
50
55
60
65
70
75
80
85
90
95
100
105
110
115
ARSFL011
UFFxa19B10
CFVCT026
EMFv018
BFACT019
EMFv021
CX661626b
CFVCT018
BFACT029
BFACT004
UFFxa20G06
CO818147
EMFn213
UFFxa03D11
EMFvi008
BFACT018
EXP1A
EMFv023
BFACT044
EMFvi109
CFVCT023
BFACT031
UFFxa19B10
CFVCT026
BFACT004
CO818147
BFACT019
UFFxa20G06
EMFv018
CFVCT032
BFACT029
CX66126b
EMFv021
ARSFL011
EMFn213
UFFxa03D11
BFACT018
EMFvi008
EXP1A
CFVCT023
EMFvi109
BFACT044
BFACT031
EMFv023
AC32
AG35
EMFv190
EMFv021
CHS
CFVCT026
UFFxa19B10
BFACT004
UFFxa20G06
CO818147
FAC001
BFACT029
ARSFl024
CX661626b
CFVCT031
ARSFL011
EPpCU2875
CFVCT031
EMFn201
CFVCT009
EPpCU9257
CX662065a
CHI
EMFn213
CFVCT019
BFACT018
EMFvi008
ARSFL099
EPpCU9223
BFACT031
CFVCT023
EMFvi109
BFACT044
EMFv023
MC45
CoMET
